# Evaluation and Optimization of a Cross-Rib Micro-Channel Heat Sink

**DOI:** 10.3390/mi13010132

**Published:** 2022-01-14

**Authors:** Haiying Chen, Chuan Chen, Yunyan Zhou, Chenglin Yang, Gang Song, Fengze Hou, Binbin Jiao, Ruiwen Liu

**Affiliations:** 1 Institute of Microelectronics of Chinese Academy of Sciences, Beijing 100029, China; chenhaiying@ime.ac.cn (H.C.); chenchuan@ime.ac.cn (C.C.); yangchenglin@ime.ac.cn (C.Y.); songgang@ime.ac.cn (G.S.); houfengze@ime.ac.cn (F.H.); jiaobinb@ime.ac.cn (B.J.); liuruiwen@ime.ac.cn (R.L.); 2 University of Chinese Academy of Sciences, Beijing 100029, China

**Keywords:** micro-channel heat sink, cross-rib, heat dissipation performance, pressure drop, aspect ratio

## Abstract

This article presents a novel cross-rib micro-channel (MC-CR) heat sink to make fluid self-rotate. For a thermal test chip (TTC) with 100 w/cm^2^, the cross-ribs micro-channel were compared with the rectangular (MC-R) and horizontal rib micro-channel (MC-HR) heat sinks. The results show that, with the cross-rib micro-channel, the junction temperature of the thermal test chip was 336.49 K, and the pressure drop was 22 kPa. Compared with the rectangular and horizontal ribs heat sink, the cross-rib micro-channel had improvements of 28.6% and 14.3% in cooling capability, but the pressure drop increased by 10.7-fold and 5.5-fold, respectively. Then, the effects of the aspect ratio (λ) of micro-channel in different flow rates were studied. It was found that the aspect ratio and cooling performance were non-linear. To reduce the pressure drop, the inclination (α) and spacing (S) of the cross-ribs were optimized. When α = 30°, S = 0.1 mm, and λ = 4, the pressure drop was reduced from 22 kPa to 4.5 kPa. In addition, the heat dissipation performance of the rectangular, staggered fin (MC-SF), staggered rib (MC-SR) and cross-rib micro-channels were analyzed in the condition of the same pressure drop, MC-CR still has superior heat dissipation performance.

## 1. Introduction

With the rapid development of integrated circuits in terms of high integration and miniaturization, the thermal design power and heat flux of electronics are increasing. It is essential to dissipate the heat accumulated inside electronics [1]. According to statistics, 55% of electronic failures are caused by heat [2,3]. In the past few decades, heat dissipation has mainly been through natural and forced convection [4,5]. Among them, natural convection does not rely on external force; fluid flows because of the temperature difference inside fluid, and the heat generated by the microelectronics is taken away and released. This method is low in cost and is only suitable for devices with low power density and no strict temperature requirements, because the heat dissipation capacity of this method is very limited [6]. Common forced convection mainly includes air-cooling and water-cooling. Air-cooling accelerates the removal of heat, which increases the velocity of the air through the fan. Additionally, water-cooling is realized by installing a liquid cooling plate on the heat source, cooperating with a heat exchanger and a pump to dissipate heat by means of fluid flow. Compared with water-cooling, the cost of air-cooling is low and the performance is stable, although the cooling capacity is limited; therefore, it is widely used in scenarios with low power density and no specific wind speed requirements. However, the heat transfer coefficient of water is 1000~15,000 W/m^2^·k in the case of forced convection, which is more than 100-fold that of air. In addition, water-cooling is quieter.

Nowadays, micro-channel heat sinks are a mainstream liquid cooling technology, with the concept first proposed by Tuckerman and Pease [7]. The technology is mainly designed to continuously take away the heat generated by microelectronics through the circulation of fluid. With satisfactory heat transfer capability, compact structure, and relatively light weight, micro-channel heat sinks can be regarded as a suitable and feasible solution to meet the heat dissipation requirements [8,9]. The key factors affecting the heat dissipation performance of the micro-channel are the type of fluid and the structure of the micro-channel. There has been much research on the shape of the micro-channel. Wang et al. [10] compared the flow characteristics and heat transfer performance of triangular, trapezoidal and rectangular micro-channels through numerical simulations. The result showed that rectangular micro-channels have the lowest thermal resistance and the best heat dissipation performance, followed by trapezoidal micro-channels and triangular micro-channels. In addition, rectangular micro-channels have the lowest thermal resistance in the aspect ratio range of 8.904–11.442. Lin et al. [11] proposed a novel wavy micro-channel heat sink that changes the wavelength and amplitude along the flow path. When the wavelength of the wave unit decreases or the amplitude increases, the wavy micro-channel heat sink has a lower thermal resistance and exhibits better overall performance. Park et al. [12] optimized the critical dimensions of wave micro-channel heat sinks with grooves, such as the width, depth and spacing of the grooves, reducing the thermal resistance and friction coefficients by 1.55% and 3%, respectively. Huang et al. [13] conducted a numerical study on the heat transfer characteristics of rectangular parallel grooves, rectangular staggered grooves, and trapezoidal staggered groove micro-channel heat sinks. The results show that under conditions of constant power, the heat transfer performance of the trapezoidal staggered groove micro-channel heat sink is the best. 

In addition, changing the flow state in the micro-channel has also attracted a large number of researchers. Ghani et al. [14] studied the fluid flow and heat transfer characteristics (MC-SCRR) of a novel sinusoidal cavity rectangular rib micro-channel heat sink. The performance of rectangular rib micro-channels (MC-RR) and sinusoidal cavity micro-channels (MC-SC) was compared and analyzed. The results show that the thermal performance of MC-SCRR is better than that of MC-RR and MC-SC. Chai et al. [15,16,17,18,19,20] studied the effect of ribs in different arrangements on the heat dissipation capacity of the micro-channel. The study found that both aligned ribs and offset ribs can increase the heat transfer area and enhance fluid disturbance. Xie et al. [21] investigated the influence of the inclined angle of the pin fins. When inserting pin fins with an inclined angle of 30° in the MCHS, intense secondary flow was generated to facilitate disturbance flow. Deng et al. [22] and Carlo et al. [23] studied the performance of double-layered micro-channel heat sinks (DL-MCHS) with different cross sections and an unequal number of micro-channels in two layers. The result shows that DL-MCHS significantly reduced wall temperature and thermal resistance, as well as pressure drop and pumping power compared with single-layered micro-channel heat sinks (SL-MCHS). In addition, when the number of micro-channels in the top layer is smaller than that in the bottom layer, there may be lower thermal resistance and better temperature uniformity. Jing et al. [24] investigated the thermal characteristics of an interleaved double-layer micro-channel heat sink with upper and lower micro-channels offset along the width direction. It was found that when the rib thickness is small, the thermal resistance increases with the increase in the offset value, and when the rib thickness is large enough, the thermal resistance decreases first and then increases with the increase in the offset value. Therefore, it will significantly enhance heat transfer but also increase pressure drop, especially aligned ribs. In summary, increasing the heat exchange area, reducing convective thermal resistance, and changing the flow state of the fluid can all improve the heat dissipation performance of the micro-channel.

This paper proposes a novel micro-channel that makes the fluid rotate. The heat dissipation performance of MC-CR was evaluated through numerical analysis and simulation; then, the influence of the aspect ratio of the micro-channel on the heat dissipation performance was analyzed. Then, the pressure drop was reduced by optimizing the inclination angle, spacing of the cross-rib, and aspect ratio of the micro-channels, which provided a reference for the optimization of the MC-CR heat sink. Finally, compared with MC-R, MC-SF, MC-SR, and MC-CR at the same pressure drop, MC-CR still had superior heat dissipation performance.

## 2. Geometrical Model and Method

**Figure 1:**
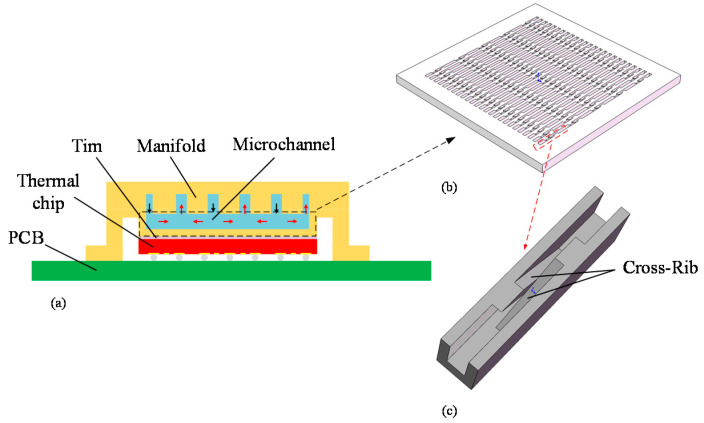
(**a**) Schematic diagram of the micro-channel and thermal chip; (**b**) dimensional schematic diagram of the MC-CR heat sink; (**c**) a basic unit in the MC-CR heat sink.

**Figure 2:**
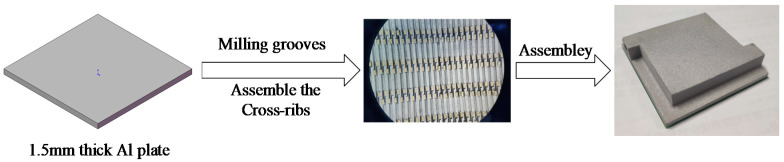
Assembly process.

In this study, the application scenario of the heat sink was an FCBGA thermal chip with a size of 25 × 25 × 0.75 mm. In order to realize the miniaturization design of the package, the heat sink was integrated inside the lid. A layer of Tim with a thickness of 100 μm was coated between the thermal test chip (TTC) and the heat sink for connection. As shown in Figure 1b, the size of the MC-CR heat sink is the same as that of the TTC, and there are 25 channels in total. Figure 1a shows a cross-sectional view of the assembly. Figure 1c shows the internal structure of MC-CR. The two crossed squares with an inclination angle of 30°, which were called cross-ribs, were added on the inner wall of the straight micro-channel. The cross-rib makes the fluid self-rotate along it, which promotes the mixing of the upper and lower parts of the fluid, and maximizes the fluid’s ability to absorb heat. Additionally, it also enhances the disturbance of the fluid, breaks the boundary layer, and significantly improves the heat dissipation. Figure 2 shows the assembly process. First, the aluminum plate was machined with a milling cutter to mill out the grooves, then the cross-ribs were manually assembled, and finally, they were glued to the manifold. However, the processing difficulty is inversely proportional to the inclination angle of the cross-ribs. Therefore, the inclination angle should be as large as possible on the premise of ensuring the heat dissipation performance.

### 2.1. Geometrical Model

**Figure 3:**
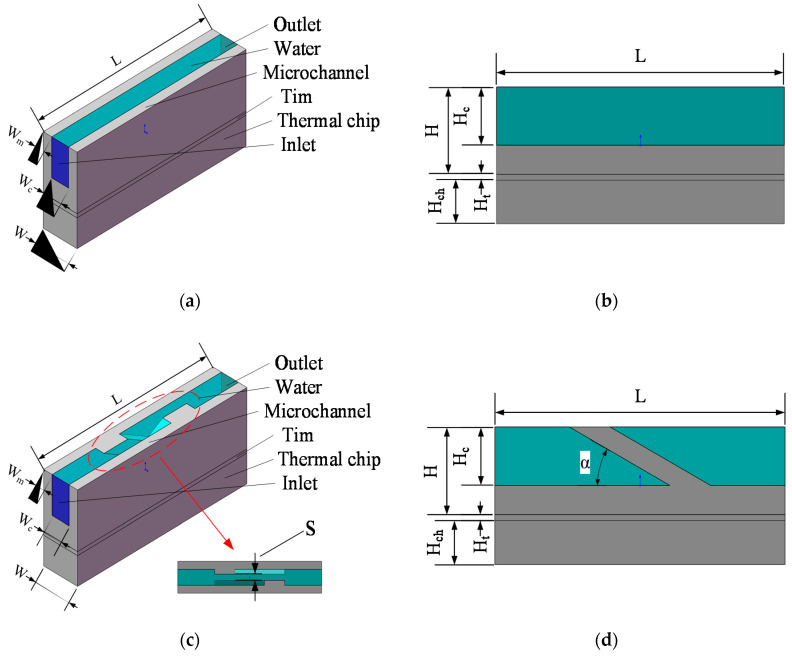

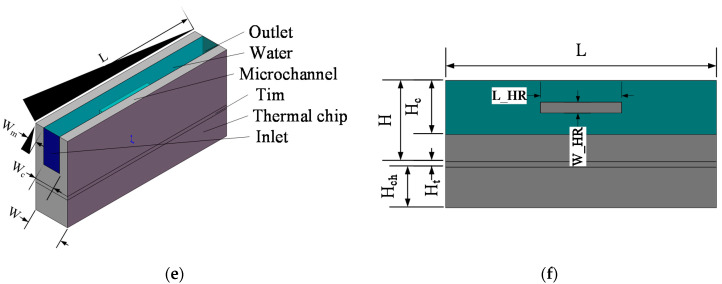
The unit models (**a**,**c**,**e**) and cross-sections (**b**,**d**,**f**) in the micro-channel heat sink: (**a**,**b**) MC-R; (**c**,**d**) MC-CR; (**e**,**f**) MC-HR.

This paper studied the influence of the micro-channel structure on heat dissipation performance, and used ANSYS fluent to solve the steady-state solution of the micro-channel heat dissipation performance. To ensure the uniqueness of variables, the width, length, depth of the heat source chip, Tim, base, and micro-channel remained the same. Figure 3 shows the basic unit model of the three micro-channels and their corresponding cross-sectional diagrams. Figure 3e shows a horizontal rib micro-channel (MC-HR) heat sink. The horizontal ribs on both sides were placed in the center of the wall and were in contact with each other, and its liquid–solid contact area was equal to MC-CR. The parameters of the positions marked in Figure 3 are tabulated in Table 1, and the ratio of the width of the fluid to the channel in the three micro-channels was 1/2 (*W_c_* = 2 *W_m_*).

### 2.2. Numerical Model and Parameter Definition

We assumed that the flow and heat transfer were in steady state, the coolant was an incompressible and Newtonian fluid, and the heat conducted from the chip was only dissipated by coolant convection; thus, heat loss to the ambient was ignored. With these assumptions, the governing equations are as follows:

Continuity equation [25]:

(1) $$\frac{\partial u_{1}}{\partial x}+\frac{\partial u_{2}}{\partial y}+\frac{\partial u_{3}}{\partial z}=0$$

Momentum equations

(2) $$\begin{array}{l} \rho (u_{1}\frac{\partial u_{1}}{\partial x}+u_{2}\frac{\partial u_{1}}{\partial y}+u_{3}\frac{\partial u_{1}}{\partial z})=-\frac{\partial p}{\partial x}+u\nabla^{2}u_{1}\\ \rho (u_{1}\frac{\partial u_{2}}{\partial x}+u_{2}\frac{\partial u_{2}}{\partial y}+u_{3}\frac{\partial u_{2}}{\partial z})=-\frac{\partial p}{\partial y}+u\nabla^{2}u_{2}\\ \rho (u_{1}\frac{\partial u_{3}}{\partial x}+u_{2}\frac{\partial u_{3}}{\partial y}+u_{3}\frac{\partial u_{3}}{\partial z})=-\frac{\partial p}{\partial z}+u\nabla^{2}u_{3} \end{array}$$

Energy equation for the coolant

(3) $$\rho c_{pl}(u_{1}\frac{\partial T}{\partial x}+u_{2}\frac{\partial T}{\partial y}+u_{3}\frac{\partial T}{\partial z})=\lambda_{l}\nabla^{2}T$$

where *u*_1_, *u*_2_ and *u*_3_ are the velocity in the *x*, *y* and *z* axis directions, respectively. ρ is the density of water.

For turbulence flow, the turbulence kinetic energy, *k*, and its rate of dissipation, *ε*, are obtained from the following transport equations:

(4) $$\frac{\partial }{\partial t}(\rho k)+\frac{\partial }{\partial x_{i}}(\rho kv_{i})=\frac{\partial }{\partial x_{j}}\left[\left(\mu +\frac{\mu_{t}}{\sigma_{k}}\right)\frac{\partial k}{\partial x_{j}}\right]+G_{k}+G_{b}-\rho \varepsilon -Y_{M}+S_{k}\;\frac{\partial }{\partial t}(\rho \varepsilon )+\frac{\partial }{\partial x_{i}}(\rho \varepsilon v_{i})=\frac{\partial }{\partial x_{j}}\left[\left(\mu +\frac{\mu_{t}}{\sigma_{\varepsilon }}\right)\frac{\partial \varepsilon }{\partial x_{j}}\right]+C_{1\varepsilon }\frac{\varepsilon }{k}(G_{k}+C_{3\varepsilon }G_{b})-C_{2\varepsilon }\rho \frac{\varepsilon^{2}}{k}+S_{\varepsilon }$$

In these equations, *G_k_* represents the generation of turbulence kinetic energy due to the mean velocity gradients, *G_b_* is the generation of turbulence kinetic energy due to buoyancy, *Y_M_* represents the contribution of the fluctuating dilatation in compressible turbulence to the overall dissipation rate. *C_1ε_*, *C_2ε_*, and *C_3ε_* are constants. *σ_k_* and *σ_ε_* are the turbulent Prandtl numbers for *k* and *ε*, respectively.

(5) $$G_{k}=-\rho \bar{v_{i}^{’}v_{j}^{’}}\frac{\partial v_{j}}{\partial x_{i}}$$

(6) $$G_{b}=\beta g_{i}\frac{\mu_{t}\partial T}{Pr_{t}\partial x_{i}}$$

(7) $$Y_{M}=2\rho \varepsilon M_{t}^{2}$$

where *β* is the coefficient of thermal expansion, *g_i_* is the component of the gravitational vector in the *i* direction, and *M_t_* is the turbulent Mach number.

In general, the Nusselt number, thermal resistance and convective heat transfer coefficient are used to evaluate the heat dissipation performance of micro-channels.

The Reynolds number (*Re*) is a dimensionless number used to characterize fluid flow and it’s defined as follows

(8) $$Re=\frac{\rho vd}{\mu }$$

(9) $$D=\frac{4A}{P}=\frac{2W_{c}H_{c}}{W_{c}+H_{c}}$$

where *v* is the flow velocity, *D* is the hydraulic diameter of the micro-channel, *μ* is the kinematic viscosity coefficient of the fluid, *A* is the cross-sectional area of the flow, and *P* is the wet perimeter.

The aspect ratio (*λ*) is defined as follows:

(10) $$\lambda =H_{c}/W_{c}$$

Water is a Newtonian fluid; therefore, the calculation equation of the pressure drop in the tube is for a turbulent Newtonian fluid, and the friction factor is defined as follows [26]:

(11) $$f=\frac{2D\Delta P}{v^{2}L\rho }=\frac{P_{o}}{Re}$$

where *L* is the characteristic length of the tube and Δ*P* is the pressure drop, which can be calculated with Equation (12)

(12) $$\Delta P=P_{in}-P_{out}$$

In laminar flow, the Poiseuille number equation in friction factor form is defined as follows [27]:

(13) $$P_{o}=24(1-1.13553\lambda +1.9467\lambda^{2}-1.7012\lambda^{3}+0.9564\lambda^{4}-0.2537\lambda^{5})$$

The Nusselt number (*Nu*) is a dimensionless number that expresses the intensity of convective heat transfer, and is defined as follows [28]:

(14) $$Nu=h_{ave}L/k_{p}$$

where *k_p_* is the thermal conductivity of the plate material and *h_ave_* is the average convective heat transfer coefficient, which is defined as follows [12]:

(15) $$h_{ave}=q/A_{c}(T_{s}-T_{io})$$

where *q* is the heat flux, *A_c_* is the area of convective heat transfer between water and solid, *T_s_* is the average temperature at the solid, and *T_io_* is the average of the inlet and outlet temperature.

The equivalent thermal resistance (*R_eq_*) is defined as [29]:

(16) $$R_{\mathrm{eq}}=R_{\mathrm{tot}}-R_{chip}-R_{tim}$$

(17) $$R_{}=\frac{L_{p}}{k\cdot A_{p}}$$

(18) $$R_{\mathrm{tot}}=\frac{T_{2}-T_{1}}{P_{\mathrm{tot}}}=\frac{T_{2}-T_{1}}{qA_{p}}$$

where *T_2_* is the average temperature of the heat source, *T_1_* is the water inlet temperature, *P_tot_* is the total power of the heat source, *R_tot_* is the total thermal resistance, *L_p_* is the thickness of the plate, and *A_p_* is the cross-sectional area of the plate perpendicular to the direction of heat flow.

### 2.3. Meshing Independence and Validation

**Figure 4:**
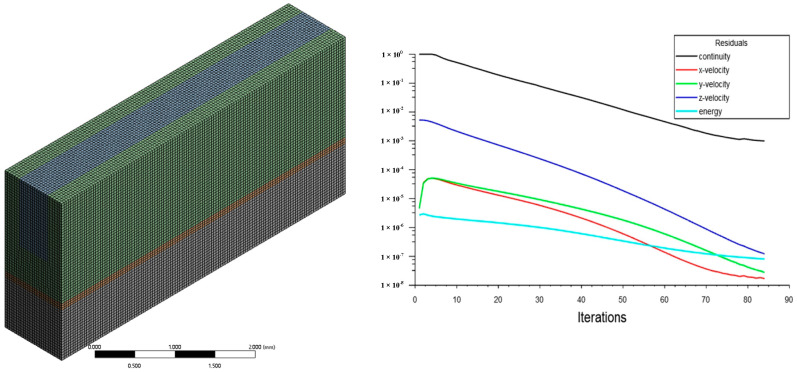
Meshed model and residuals.

Eliminating the influence of meshing on the results ensures the reliability of the simulation results. Taking the MC-R heat sink as an example, the average temperature of five meshing methods is compared. Table 2 lists the minimum element size, the total number of grids, and the average temperature of the heat source for the five meshing methods. It can be seen that with the refinement of the grid, the simulation results are more accurate and more consistent with the theoretical values. However, the difference between the results of Method Ⅳ and Method Ⅴ is 0.87%. Therefore, in order to ensure accurate simulation results and higher calculation efficiency, the subsequent simulations all used Method Ⅳ (the minimum unit size was 0.04 mm and the number of grids is 187,500). Figure 4 shows the mesh model and residual curve of Method Ⅳ.

**Figure 5:**
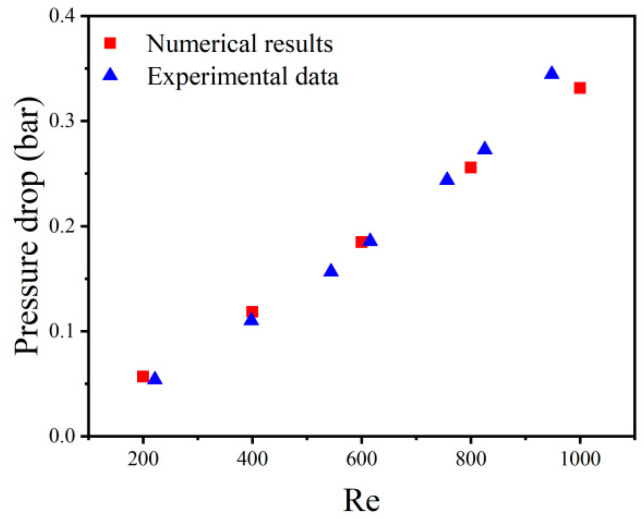
Comparison between numerical and experimental data.

To verify the reliability of numerical results, the numerical results of the present method were compared with experimental data [30]. As shown in Figure 5, the numerical results agreed well with the experimental data. Additionally, the maximum error was less than 5% in different Reynolds numbers, which is within the acceptable range.

### 2.4. Setup and Boundary Conditions

At a reference temperature of 300 K, the kinematic viscosity of water is 8.937 × 10^−5^ m^2^/s [31]. The flow velocity was relatively uniform in the MC-R heat sink, and the Re was calculated as 2237.9 (Equations (8) and (9)); therefore, the laminar flow model was used to solve it. However, in the heat sink with special-shaped structures of MC-CR and MC-HR heat sinks, the fluid in the channels no longer maintained an orderly stratified flow, so turbulence models were used to solve it. In order to simplify the simulation setup, it was assumed that the microelectronic chip heated up uniformly. The details of the boundary conditions are as follows,

1. The inlet adopted velocity-inlet flow rate was 2 m/s;
2. The heat flux of the TTC was 100 w/cm^2^;
3. The material of fluid was water (0.6 W/m·K), the material of TTC was Si (148 W/m·K), the material of micro-channel was Al (202.2 W/m·K), and the thermal conductivity of Tim was 5 W/m·K;
4. In the adopted pressure-outlet, the pressure was set to atmospheric pressure.

## 3. Result and Discussion

### 3.1. Effect of Ribs

**Figure 6:**
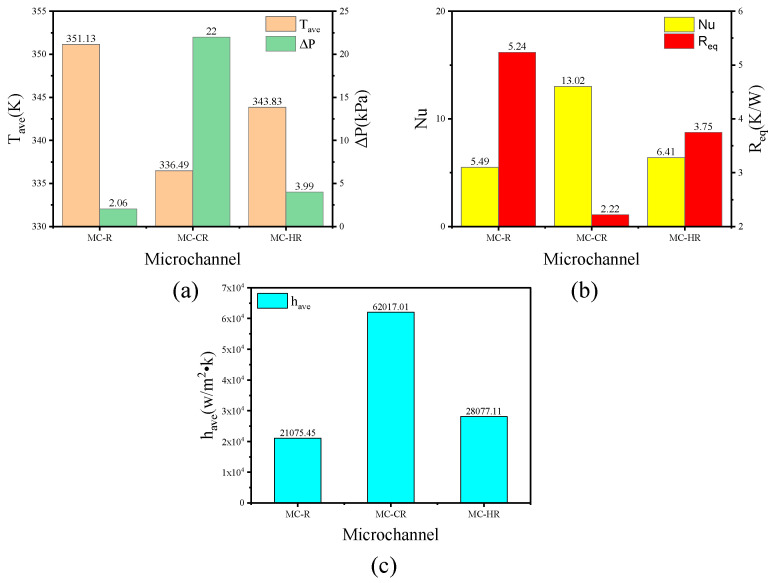
The performance of MC-R, MC-CR and MC-HR when the velocity was 2 m/s. (**a**) The average temperature and the pressure drop; (**b**) the Nu and the equivalent thermal resistance; (**c**) the average convective heat transfer coefficient.

In the same conditions, the heat dissipation performance of the micro-channel heat sink was characterized by the temperature of the heat source and pressure drop. It can be seen from Figure 6a that the temperatures of the TTC in the MC-CR and MC-HR were 14.64 K and 7.3 K lower than that in the MC-R, respectively. Therefore, the performance improvement in MC-HR was 50% that of MC-CR. The MC-CR and MC-HR had the same liquid–solid contact area, but the fluid was still in laminar flow. Therefore, the effects of flow disturbance and contact area on the heat dissipation performance of MC-CR are equal.

**Figure 7:**
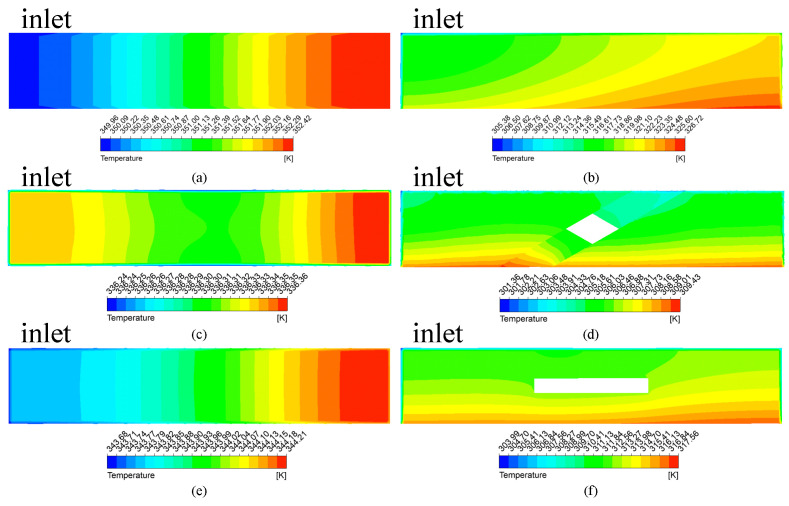
The temperature contours on the horizontal (**a**,**c**,**e**) and longitudinal (**b**,**d**,**f**) cross-sections: (**a**,**b**) MC-R; (**c**,**d**) MC-CR; (**e**,**f**) MC-HR.

As shown in Figure 7a,c,e, the maximum temperature difference of MC-CR is 0.12 K, and that of MC-R is 2.46 K. Therefore, MC-CR has excellent cooling capacity and heat dissipation uniformity. Figure 7b,d,f shows the sidewall temperature distribution of MC-CR and MC-R heat sinks, respectively. It can be seen that in the direction of flow, the thermal boundary layer of MC-R gradually thickens (Equations (19) and (20)), which will greatly reduce the heat conduction at the contact interface; the tendency of the thermal boundary layer of MC-CR to thicken is broken due to the disturbance of the fluid. At the same time, the cross-ribs make the fluid flow upward after absorbing heat, and the cold fluid on it will be replenished in time, so that the upper and lower fluids can fully absorb the heat. For MC-HR, although the horizontal ribs did not make the boundary layer thinner, they played a balanced role, which reduced the temperature difference of the TTC to 0.53 K.

The thickness of the boundary layer (*δ*(*x*)) is defined as follows:

(19) δ1(x)∝x/Rex

(20) δ2(x)∝x/ 5Rex

where *δ*_1_(*x*) and *δ*_2_(*x*) are the boundary layer thickness of the laminar and turbulent, respectively; *x* is the distance of fluid flow, and Re_x_ is the Reynolds number at *x*.

**Figure 8:**
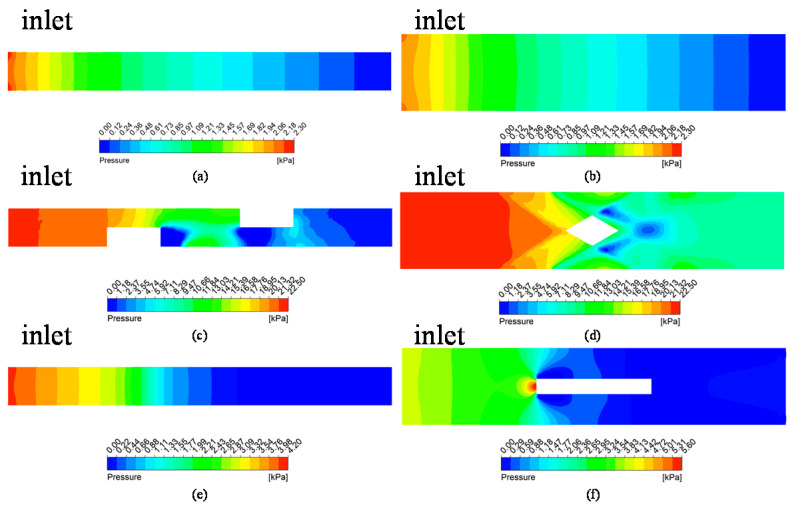
The pressure drop contours on the horizontal (**a**,**c**,**e**) and longitudinal (**b**,**d**,**f**) cross-sections: (**a**,**b**) MC-R; (**c**,**d**) MC-CR; (**e**,**f**) MC-HR.

Figure 6a illustrates that the pressure drop of MC-CR is 10.7-fold larger than that of MC-R, and the MC-HR is 1.94-fold larger than that of MC-R. It can be seen from Figure 8a–f that the pressure is mainly concentrated where the rib is perpendicular to the flow direction. Therefore, the cross-ribs severely block the fluid flow and forcibly change the flow direction, which is one of the reasons for increasing the pressure drop. This also shows that the improvement in MC-CR’s heat dissipation performance comes at the expense of pressure drop. However, this is in contrast to the concept of energy saving, so this article provides some ideas to reduce the pressure drop in Section 4.

**Figure 9:**
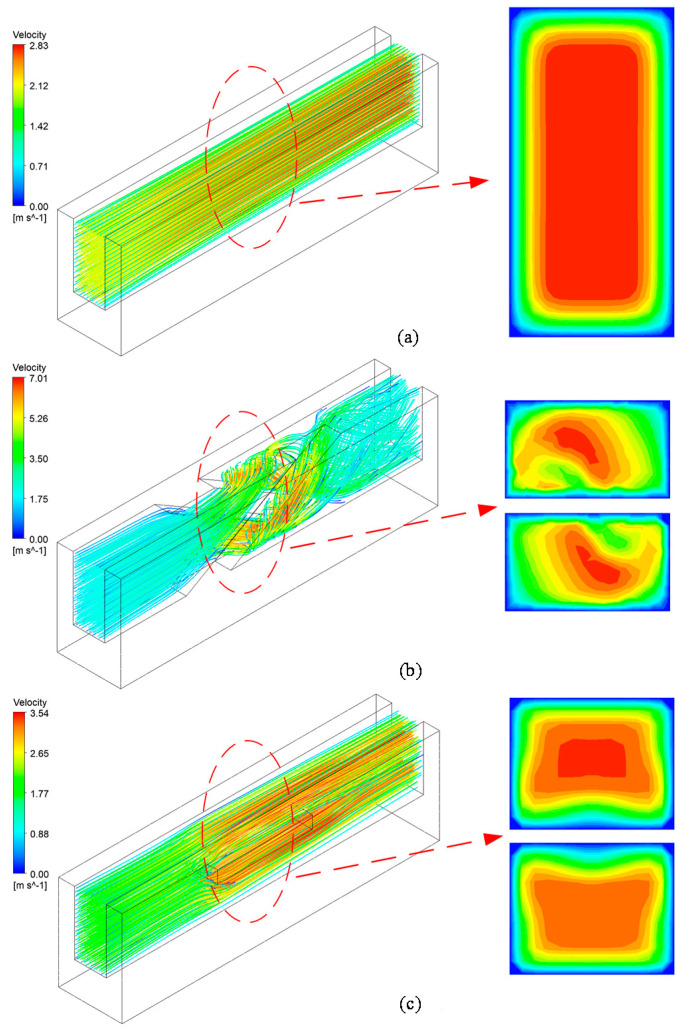
The streamline and velocity contours. (**a**) MC-R; (**b**) MC-CR; (**c**) MC-HR.

Figure 9 shows the streamlines of the fluids and velocity contours in the MC-R, MC-CR, and MC-HR heat sinks. It can be seen that the fluid flow in the MC-R has obvious stratification. The velocity of the center is the largest. The position near the wall is the velocity boundary layer in which the flow velocity is almost 0, due to the viscous force of the fluid, which cannot be ignored. The thermal conductivity of water is 0.6 W/m·K; therefore, the boundary layer would hinder the heat conduction. In the MC-CR, the cross-ribs structure makes the fluid self-rotate, which greatly disrupts the tendency of flow rate stratification and increases the flow rate and disturbance. Both of these methods increase the *h_ave_* and *Nu*, and thin the boundary layer. As shown in Figure 6b, the MC-CR has the largest *Nu* and the smallest *R_eq_*. In the MC-HR, although the horizontal ribs increase the heat transfer area and *h_ave_* (Figure 6c), they do not improve the flow rate stratification (Figure 9c). Above and below the horizontal ribs, the flow velocity boundary layer still exists.

### 3.2. Effect of Aspect Ratio λ on Heat Dissipation Performance

In order to further study the relationship between the heat dissipation performance and the *v*, *W_c_* and *H_c_*, this study designed eight MC-CRs with different aspect ratios (Table 3), and the heat dissipation performance of eight MC-CRs with different aspect ratios was analyzed at flow rates of 1 m/s, 2 m/s and 3 m/s.

**Figure 10:**
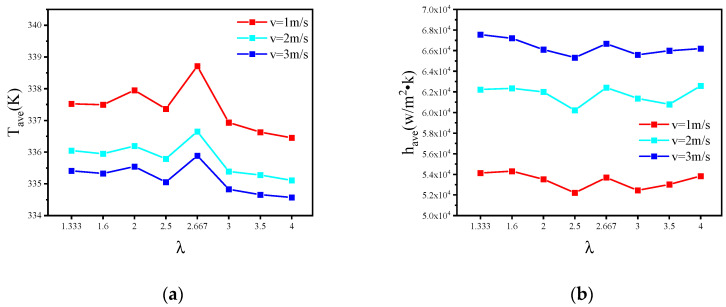

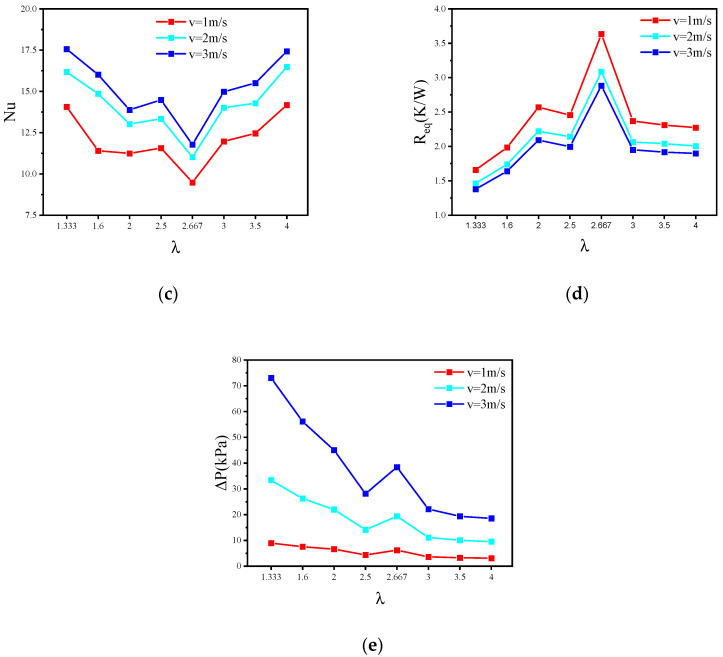
The heat dissipation performance of MC-CR with different aspect ratios. (**a**) The average temperature; (**b**) the average convective heat transfer coefficient; (**c**) Nusselt number; (**d**) the equivalent thermal resistance; (**e**) the pressure drop.

Figure 10 shows the *T_ave_*, *h_ave_*, *Nu*, *R_eq_* and Δ*P* at different flow rates. As the flow rate increased, the improvements in heat dissipation performance of the microchannel tended to be saturated, but the pressure drop greatly increased. In addition, the thickness of the ribs also changed with the aspect ratio for MC-R. Therefore, it needed to be compared in groups. In the first, second, third, and fifth experiments, *H_c_* remained constant, *W_c_* decreased, *L* decreased, the *λ* increased, and the fluid–solid contact area decreased. This phenomenon weakened the turbulence of the fluid and increased the heat transfer coefficient and Nusselt number, which also indicates that the heat convection was weakened; therefore, the heat dissipation performance became worse. In addition, *D* decreased, the pressure drop should have increased, but for MC-CR, the pressure drop became smaller with the resistance to the fluid dropping due to the thinner thickness of the cross-ribs. In the fourth, sixth, seventh, and eighth experiments, *W_c_* remained constant, *H_c_* increased, *L* increased, *λ* increased, and the fluid–solid contact area increased. This phenomenon enhanced the turbulence of the fluid and increased the heat transfer coefficient and Nusselt number. Therefore, the heat dissipation performance was improved. In addition, D increased, so the pressure drop decreased. In summary, when *λ* was 4 and the flow rate was 2 m/s, the MC-CR had better heat dissipation performance and a lower pressure drop.

## 4. Parameter Optimization

This section focuses on optimizing the above-mentioned MC-CR. Even if MC-CR has better heat dissipation performance, its pressure drop must be reduced as much as possible. Therefore, the inclination angle, *α*, and spacing of the cross-ribs and the aspect ratio, *λ*, of the micro-channel were optimized.

When the flow velocity, tube length and fluid density are constant, reducing the inclination angle of the rifling structure and increasing the spacing will decrease the pressure drop (Equation (11)) because of the reduced friction coefficient; the effect of the aspect ratio on pressure drop was analyzed in the previous section, and when *λ* was 4, the pressure drop was the smallest.

### 4.1. The Inclination Angle (α) of the Cross-Ribs

Figure 8a–f shows that the pressure drop was concentrated where the rib was perpendicular to the flow direction. Therefore, keeping the other parameters consistent, this section analyzes the pressure drop and the average temperature of the TTC when the inclination angles, *α*, of the cross-ribs are 45°, 40°, 35°, 30°, and 25°.

**Figure 11:**
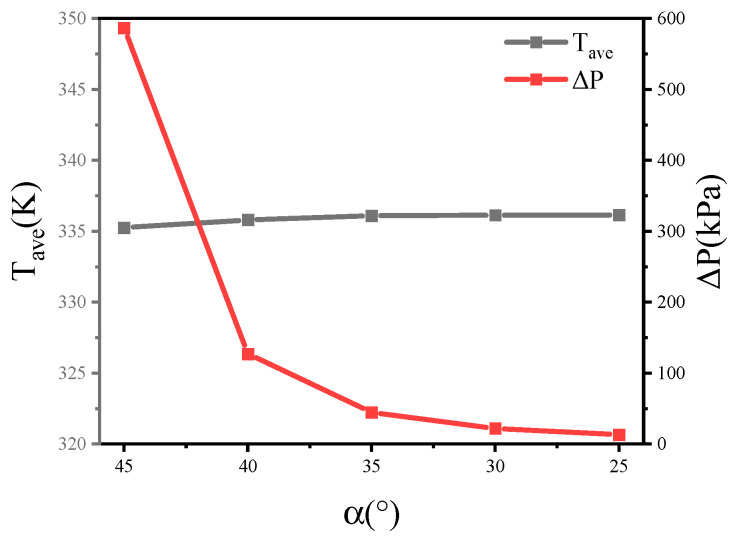
The average temperature and pressure drop of the heat source in different inclination angles of the cross-ribs.

The resistance coefficient of the fluid in the MC-CR heat sinks is derived from the resistance of the cross-ribs structure and the wall friction. Equation (11) illustrates that the resistance coefficient is proportional to the pressure loss. Figure 11 shows that as the inclination angle, *α*, decreases, the heat dissipation performance becomes worse, and the pressure drop increases. The inclination angle, *α*, was reduced from 45° to 25°, and the average temperature rose by 0.3% in total; therefore, the change in inclination angle, *α*, has little effect on the heat dissipation performance. However, when the inclination angle, *α*, was reduced from 45° to 25°, the pressure drop was reduced by 97.7% in total, which is significant.

### 4.2. The Spacing (S) of the Cross-Rib

Through the preliminary simulation, it was found that fluid rotation can also be guaranteed in the condition where cross-ribs structure does not make contact. Therefore, in the condition that other parameters remain constant, this section analyzes the pressure drop and the average temperature of the heat source when the cross-rib spacing, *S*, is 0 mm, 0.05 mm, 0.1 mm, 0.15 mm, and 0.2 mm.

**Figure 12:**
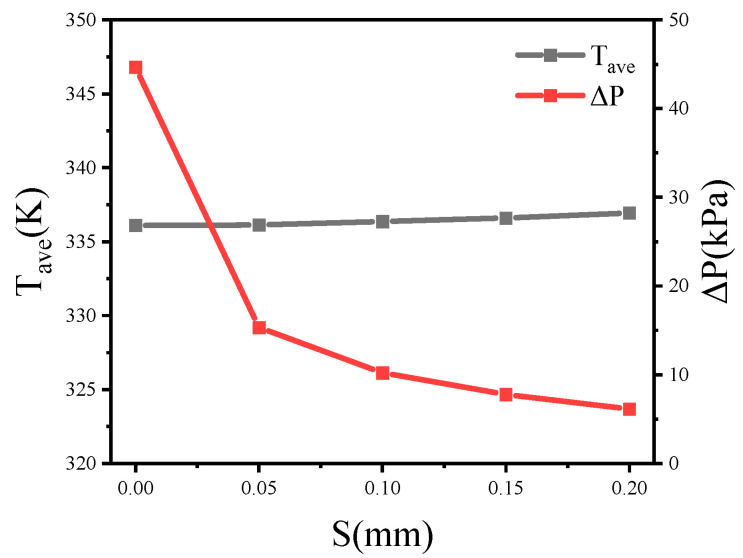
The average temperature and pressure drop of the heat source with different spacing of the cross-ribs.

It can be seen from the streamline (Figure 9) that larger spacing in the cross-rib structure allows part of the fluid to flow directly through the middle, which reduces the area where the fluid is blocked, therefore reducing the pressure drop. Figure 12 shows that as the spacing was increased from 0 mm to 0.2 mm, the pressure drop was reduced by 86.3% in total. However, at the same time, the increase in spacing also slowed down the rotation of the fluid, causing an average temperature increase of 0.84 K.

Due to process limitations, as *α* decreases, it becomes more difficult for the milling cutter to mill the groove. Therefore, without affecting the heat dissipation performance, in the condition where *α* was 30°, the *S* was 0.1 mm, and *λ* was 4, the pressure was reduced from 22 kPa to 4.5 kPa.

### 4.3. Effect of ribs in Same Pressure Drop

**Figure 13:**
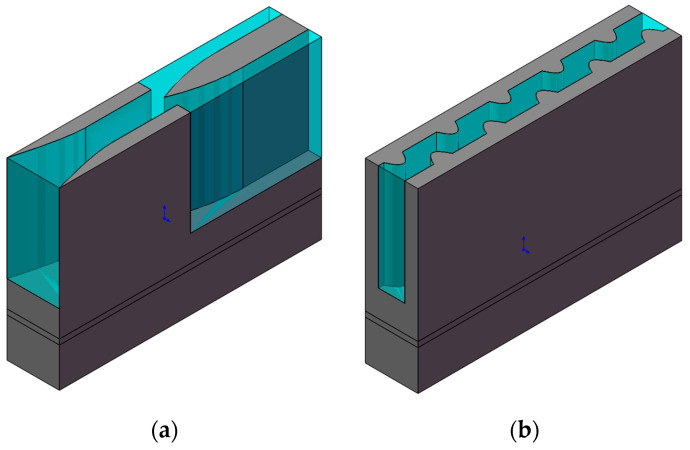
The unit models of the micro-channel heat sinks: (**a**) MC-SF; (**b**) MC-SR.

At the same inlet flow rate, comparison between MC-CR, MC-R, MC-SF and MC-HR showed that MC-CR had better heat dissipation performance. However, that superior performance comes with the expense of high pressure drop in the structure. Therefore, this section compares the heat dissipation performance of MC-CR, MC-SF and MC-SR heat sinks with the same pressure drop (4.5 kPa). Figure 13a shows the unit model of the MC-SF, which diverted the water continuously to reduce the thickness of the boundary layer. As shown in Figure 13b, the MC-SR is a staggered rib micro-channel, as proposed by Chai et al. [15].

**Figure 14:**
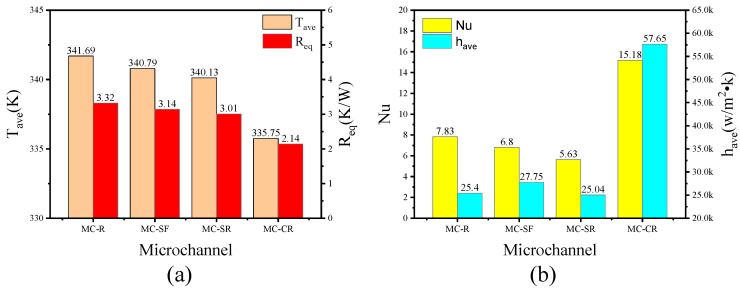
The performance of MC-R, MC-SF, MC-SR, and MC-CR when the pressure drop is 4.5 kPa. (**a**) The average temperature and the equivalent thermal resistance; (**b**) the Nu and average convective heat transfer coefficient.

It can be seen from Figure 14a that the MC-CR still has obvious advantages at the same pressure drop. Compared with MC-R, the cooling performance of MC-CR increased by 14.25%, whereas MC-SF and MC-SR increased by 2.16% and 3.74%, respectively. Figure 14b shows that the *Nu* of MC-R is larger than that of MC-SF and MC-SR, but its heat dissipation performance is worse. The reason is that the heat dissipation performance is determined by both heat conduction and convection, and the boundary layer with poor thermal conductivity of MC-R makes heat conduction weaker. One of the advantages of MC-CR is that the boundary layer is well broken; thus, the heat dissipation performance has been greatly improved.

## 5. Conclusions

In this paper, a new cross-rib micro-channel heat sink has been proposed. The heat dissipation performance of the MC-CR was studied through numerical analyses, and the key parameters were optimized. The conclusions are as follows:

(1) Compared with MC-R, the heat dissipation performance of MC-CR increased by 28.6%, and the pressure drop increased by 10.7-fold. Comparing with MC-HR, which has the same liquid–solid contact area, the heat dissipation performance increased by 14.3%. Therefore, the effects of fluid disturbance and contact area on the heat dissipation performance of MC-CR are equal;
(2) Increasing the aspect ratio will increase the liquid–solid contact area, which is conducive to improving the heat dissipation performance. However, for MC-R, the thickness of the ribs will also change with the aspect ratio. Therefore, in the condition with constant *H_c_*, the heat dissipation performance is inversely proportional to the aspect ratio; when the *W_c_* is constant, the heat dissipation performance is proportional to the aspect ratio;
(3) As the inclination angle, *α*, decreases, the average temperature rises by 5.7% overall, although the overall pressure drop is reduced by 97%. Meanwhile, the heat dissipation performance deteriorates by 5.6%, and the pressure drop is reduced by 86.3% as the spacing, *S*, increases. The heat dissipation performance is increased by 6.6%, and the pressure drop is reduced by 78.7% with the spacing, *S*, increases. In the condition where *α* = 30°, *S* = 0.1 mm, and *λ* = 4, the pressure drop is 4.5 kPa;
(4) With the same pressure drop, the cooling performance of MC-CR increased by 14.25%, and the convection heat transfer coefficient increased by 2.27-fold compared with MC-R. The cooling performance of MC-CR increased by 10.51%, and the convection heat transfer coefficient increased by 2.3-fold compared with MC-SR.

## Figures and Tables

**Table 1:** The size parameters of the marked position on the three views in Figure 3.

Parameter	Dimension	Parameter	Dimension
*L*	5 mm	*H*	1.5 mm
*W*	1 mm	*H_ch_*	0.75 mm
*W_c_*	0.5 mm	*H_c_*	1 mm
*W_m_*	0.25 mm	*H_t_*	0.1 mm
*W_HR*	0.2 mm	*L_HR*	1.5 mm
*α*	30°	*S*	0

**Table 2:** Five meshing methods and simulation results.

Method	I	II	III	IV	V
Minimum unit size (mm)	0.1	0.08	0.06	0.04	0.02
Grid number	11,500	23,751	55,029	187,500	1,504,500
Temperature (K)	360.771	356.880	353.179	351.128	351.576

**Table 3:** Detailed information about the aspect ratio of MC-CR.

*No*	*H_c_*	*W_c_*	*λ*(*H_c_/W_c_*)
1	1	0.75	1.333
2	1	0.625	1.6
3	1	0.5	2
4	1.25	0.5	2.5
5	1	0.375	2.666
6	1.5	0.5	3
7	1.75	0.5	3.5
8	2	0.5	4

## Data Availability

The data presented in this study are available on request from the corresponding author. The data are not publicly available due to privacy.
